# *Ehretia asperula* from Vietnam: Evaluation of genetic diversity, bioactive compound, and micropropagation

**DOI:** 10.5511/plantbiotechnology.25.1009a

**Published:** 2026-03-25

**Authors:** Huong Thi Trinh, Hang Thu Thi Nguyen, Truc Thanh Phan To, Nam Van Hoang, Loan Quynh Le, Dung Hoang Nguyen, Trang Huyen Thi Nguyen, Thuy Kim Thi Dang, Giap Dang Do, Tuan Trong Tran

**Affiliations:** 1 Ho Chi Minh City University of Industry and Trade, HCM City 700000, Vietnam; 2 Plant Biotechnology and Agriculture Department, Institute of Life Sciences, VAST, HCM City 700000, Vietnam; 3 Microbiology Department, Institute of Life Sciences, VAST, HCM City 700000, Vietnam

**Keywords:** 2-isopentenyladenine, *Ehretia asperula*, ITS sequence, micropropagation, rosmarinic acid

## Abstract

*Ehretia asperula* Zoll. et Mor. (“Xạ đen”) is a valuable medicinal plant widely used in traditional Vietnamese medicine due to its rich content of bioactive compounds. This study investigated the phytochemical composition, antioxidant activity, and genetic diversity of samples collected from three regions in Vietnam. ITS marker and methods for quantifying secondary metabolites were used to assess the genetic diversity and chemical composition of *E. asperula* across different collection sites. Although ITS1 sequence analysis showed no significant genetic variation among accessions, there were notable differences in secondary metabolite content. Plants from Hoa Binh province contained the highest levels of total phenolic, flavonoids, and rosmarinic acid, followed by those from Dong Nai and Vinh Phuc provinces. Among the tested solvents, an ethanol-water (30 : 70, v/v) proved most effective for extracting the targeted compounds. In addition, a micropropagation protocol was successfully established using nodal explants from the plants collected from Hoa Binh province. Optimal surface sterilization was achieved with 0.1% HgCl_2_ for 16 min. Shoots were most efficiently regenerated from nodal explants on MS medium supplemented with 3.0 mg l^−1^ kinetin. Shoot proliferation was most effective on MS medium supplemented with 1.5 mg l^−1^ 2-isopentenyladenine, while root induction reached 100% efficiency on MS medium containing 1.0 mg l^−1^ indole-3-butyric acid. These findings highlight the potential of *E. asperula* as a sustainable natural antioxidant source and support its continued use in traditional medicine.

## Introduction

Medicinal plants are vital to human health as they produce diverse bioactive compounds. These valuable constituents are found in both wild and cultivated species. In many cases, traditional medicinal plants serve dual purposes, functioning not only as therapeutic agents but also as components of daily diets. Vietnam has a rich diversity of traditional medicinal plants, represent a valuable reservoir of bioactive natural products. In recent years, the exploration of these compounds, particularly those used in traditional Vietnamese medicine, has attracted considerable scientific interest among domestic researchers. Such investigations have identified of numerous novel biologically active compounds with potential value in modern drug discovery (Giang and Otsuka 2018). Medicinal plants thus constitute a fundamental component of Vietnam’s indigenous medical systems and are widely regarded as part of the nation’s traditional knowledge and cultural heritage (Duong-Trung et al. 2019). Vietnam is recognized as one of the world’s top ten biodiversity centers and ranks 16th globally regarding genetic resource diversity. Among these resources, many medicinal plant genetic resources are utilized for disease prevention and treatment. It is estimated that there are approximately 3,948 medicinal plant species used for therapeutic purposes in Vietnam, accounting for about 33% of the country’s 12,000 plant species in the flora of Vietnam (Nguyen 2011; Pham 1999; Vo 1997). Vietnam’s known medicinal plant species represent approximately 11% of the estimated 35,000 medicinal plant species worldwide. In addition, Vietnam’s humid tropical monsoon climate and diverse topography contribute to its rich diversity of plant species with medicinal value. Several medicinal plant species have been extensively studied and widely used in Vietnam, including *Panax vietnamensis* (Ngoc Linh ginseng), *Eurycoma longifolia* (Tongkat ali), *Morinda officinalis*, and *Gynostemma pentaphyllum*.

In Vietnamese traditional medicine, the plant species commonly known as ‘Xạ đen’ (*Ehretia asperula*), belonging to the *Boraginaceae* family, is widely used for the treatment of various ailments, including hepatitis, gastritis, and skin abscesses, and is valued for its antioxidant and anti-inflammatory properties. Nguyet et al. (2018) isolated six compounds from the ethyl acetate fraction of *E. asperula* stem, including daucosterol, coniferaldehyde, methyl caffeate, 9′-methoxydehydrodiconiferyl alcohol, oresbiusin B, and vanillic acid. Among them, methyl caffeate exhibited potent cytotoxic activity against Hep-G2, HeLa, and MCF-7 cancer cell lines. In addition, Le et al. (2021) isolated ten compounds from the ethanol extract of *E. asperula* leaves and evaluated their ability to prevent retinal cell death caused by oxidative stress. The study further demonstrated that rosmarinic acid (RosA) and methyl rosmarinate protect retinal cells against oxidative damage and other harmful agents. Duc et al. (2024) conducted a study to evaluate the antioxidant activity of seven compounds isolated from this species, including caffeic acid, (−)-loliolide, kaempferol, astragalin, nicotiflorin, rutin, and daucosterol. The results showed that all these compounds exhibited antioxidant activity (Duc et al. 2024). In the same year, Le et al. (2024) demonstrated the anti-inflammatory activity of *E. asperula* leaves by evaluating the inhibitory effect of its leaf extract on the cyclooxygenase-2 (COX-2) enzyme (Le et al. 2024). According to the study by Piao et al. (2024), RosA protects the skin from UVB-induced damage by activating AKT and ERK signalling pathways, which regulate NRF2 signalling and enhance GSH biosynthesis. Therefore, treatment with RosA may represent a promising strategy for protecting the skin against oxidative damage caused by UV_B_ radiation. Leaf extracts of *E. asperula* have also been analyzed for total phenolic content (TPC), total flavonoid content (TFC), and antidiabetic activity, demonstrated by their inhibitory effects on the enzymes α-glucosidase and α-amylase (Duc et al. 2025).

In 2016, our study identified the presence of RosA in *E. asperula* leaves collected from Hoa Binh province using Nuclear Magnetic Resonance spectroscopy (Tuan et al. 2016). RosA is a common phenolic compound in plant species belonging to the *Boraginaceae* and *Lamiaceae* families (Petersen et al. 2009). It is typically present in various species of *Boraginaceae* and in members of the subfamily *Nepetoideae* within the *Lamiaceae* family, such as *Rosmarinus officinalis*, *Perilla* spp, and *Salvia officinalis* (Petersen and Simmonds 2003). In addition, RosA has also been identified in higher plants such as ferns from the *Blenchnaceae* family, in some lower plants such as hornworts, and monocotyledonous plants, including seagrasses from the *Zosteraceae* family and members of the *Potamogetonaceae* family. This distribution indicates the widespread presence of RosA in plants (Petersen and Simmonds 2003). The occurrence of RosA across diverse plant species highlights biodiversity and provides a strong foundation for research and development of natural products.

In addition to studies on the bioactive compounds of this species, research on propagation methods and plant tissue culture has also been conducted. Our earlier research successfully regenerated of in vitro shoots from stem explants of *E. asperula* collected in Hoa Binh province, Vietnam (Tuan et al. 2016). Similarly, the study conducted by Tram et al. (2018) studied the influence of light-emitting diode (LED) lighting on the growth, antioxidant potential, and phenolic content of in vitro *E. asperula*. After four weeks of culture, plants exposed to growth light LEDs exhibited greater height, higher biomass accumulation, elevated phenolic levels, and more potent antioxidant activity compared with plants under other lighting treatments. Similarly, Le et al. (2019) conducted in vitro culture of *E. asperula* nodes. Their results indicated that the optimal mineral medium for shoot induction from stem explants was half-strength MS supplemented with 0.1 mg l^−1^ BA. Root formation was most effective on half-strength MS medium supplemented with 0.25 mg l^−1^ IBA.

In Vietnam, this species is mainly found in the mountainous areas of the northern region (Nguyen et al. 2017). However, in recent years, due to its high medicinal value, local communities have increasingly cultivated it in various parts of the country. In addition, seven *Ehretia* species have been recorded in Vietnam (Hoang 2010), among which *E. asperula* exhibits considerable morphological similarity to other *Ehretia* species, making species identification and cultivation practices more complex. Assessing the genetic diversity of *E. asperula* from different regions of Vietnam is essential for determining the species’ origin and ensuring accurate identification. In the Nguyen et al. (2017) study, three molecular markers, ITS1, trnL-trnF, and matK, were employed to assess the phylogenetic position of *E. asperula*. Based on ITS1 sequence analysis, *E. asperula* was classified within the Ehretia I clade and identified as a close relative of *E. resinosa*. Furthermore, the ITS1 sequence was recommended as an effective DNA marker for accurately identifying *E. asperula*.

Therefore, the application of biotechnology, particularly DNA barcoding for species identification, and the development of large-scale in vitro culture protocols represent essential research directions. Tissue culture techniques not only contribute to the conservation and development of medicinal plant genetic resources but also enable the production of stable cell biomass for use in the pharmaceutical and cosmetic industries. In addition, identifying source materials, assessing genetic stability, and evaluating compound content in both mothers and in vitro plants play a crucial role in improving biomass quality for propagation purposes and long-term applications. This study focuses on assessing the genetic stability and phytochemical content of *E. asperula* collected from three different sources, with the aim of selecting high-quality mother plants for establishing an effective micropropagation protocol for this species.

## Materials and methods

### Plant material

*E. asperula* plants were collected from three regions, including Hoa Binh, Vinh Phuc, and Dong Nai, Vietnam. These plants were subsequently cultivated for about 2 years at the nursery of the Institute of Life Sciences, Vietnam Academy of Science and Technology, in Ho Chi Minh City, Vietnam. For this study, *E. asperula* plants collected from different regions were temporarily labelled as HB (Hoa Binh), VP (Vinh Phuc), and DN (Dong Nai).

Leaves from mature plants were collected and used as raw materials for experiments on quantifying secondary metabolites (including TPC, TFC, and RosA content) and antioxidant activity. In addition, stem segments were used as explants in shoot regeneration and proliferation experiments to develop a micropropagation method for this species.

### Medium culture

The culture medium used was Murashige and Skoog (MS) basal medium (Murashige and Skoog 1962), supplemented with 30 g l^−1^ sucrose and 8 g l^−1^ agar. Depending on the experiment, various plant growth regulators (KIN, BA, 2-iP, NAA, and IBA) were added at different concentrations. All media were adjusted to a pH of 5.7–5.8 and sterilized by autoclaving at 121°C and 1 atm pressure for 20 min.

### Chemicals

Rosmarinic acid (≥98%, UPLC grade) was used as the standard and obtained from Merck Sigma. Other chemicals used in this study included ethanol (Chemsol, Vietnam), sodium carbonate (Na_2_CO_3_, Merck, Germany), gallic acid (Merck), Folin–Ciocalteu’s phenol reagent (Merck), and 2,2-diphenyl-1-picrylhydrazyl (DPPH, Sigma-Aldrich). In addition, formic acid (Xilong, China), methanol (≥99.9%, Merck), and acetonitrile (Merck) were used for chromatographic analysis.

KIN, BA, 2-iP, NAA, and IBA were purchased from Duchefa Biochemie (The Netherlands).

### Culture conditions

All culture vessels were illuminated with white LED lamps (Philips, Vietnam) under a 12-h photoperiod at 40 µmol m^−2^ s^−1^ light intensity. The average temperature in the culture room was maintained at 25±2°C, with a relative humidity of 55–60%.

### Experimental designs

#### Assessment of genetic diversity of *E. asperula*

*E. asperula* leaves were obtained from three different sources for comparative analysis. Total genomic DNA was extracted using a modified CTAB protocol adapted for the efficient isolation of DNA from *E. asperula* leaves (Doyle and Doyle 1987). The genomic DNA of *E. asperula* were quantified using the SpectraMax QuickDrop spectrophotometer. The absorbance values at 260 and 280 nm wavelengths were measured, and the DNA purity was assessed based on the OD_260_/OD_280_ ratio. Total DNA samples with an OD_260_/OD_280_ ratio ranging from 1.8 to 2.0 were considered pure (free from protein contamination) and were selected for PCR amplification using universal primer pairs (ITS-AB-101 (ACGAATTCATGGTCCGGTGAAGTGTTCG) and ITS-AB-102 (TAGAATTCCCCGGTTCGCTCGCCGTTAC) (Nguyen et al. 2017). The PCR products were analyzed by electrophoresis on a 1.2% agarose gel at 100 V for 30 min, and the results were visualized and documented using the Analytik Jena™ UVP ChemStudio PLUS gel documentation system. The amplified products were sequenced using the Sanger method in both directions. To complete this study’s dataset, DNA sequences along with their corresponding GenBank accession numbers were retrieved, including 21 sequences of *Ehretia* species and one sequence designated as an outgroup (Table 1). The sequences were edited using MEGA X software and checked for discrepancies. Sequence similarity and coverage were evaluated by comparing the DNA sequences with those in the NCBI database using the BLAST tool. A phylogenetic tree was constructed using MEGA X, IQ-Tree and ITOL with a bootstrap value of 1000.

**Table 1:** Table 1. List of species and ID numbers of ITS1 sequences on GenBank (NCBI).

Species	ID number	Species	ID number
*Ehretia asperula*	KY320205	*Ehretia obtusifolia*	AY331401
*Ehretia acuminata*	AF385799	*Ehretia macrophylla*	AF385802
*Ehretia anacua*	AF385796	*Ehretia microphylla*	AY463160
*Ehretia aquatica*	AF385791	*Ehretia monopyrena*	AF385792
*Ehretia cortesia*	AY463159	*Ehretia resinosa*	AY463161
*Ehretia coerulea*	KF673249	*Ehretia rigida*	AF385789
*Ehretia cymosa*	AF385790	*Ehretia saligna*	AF385786
*Ehretia laevis*	AF385787	*Ehretia tinifolia*	AF385793
*Ehretia latifolia*	AF385797	*Ehretia wallichiana*	AY331402
*Ehretia longiflora*	AY331400	*Bourreria succulenta*	AF385776

#### Assessment of total phenolic content, flavonoid content, rosmarinic acid, and antioxidant activity of *E. asperula*: Preparation of plant material and leaf extract

*E. asperula* leaves were collected from the nursery, then dried at 45°C until reaching a moisture content of approximately 6%. The dried leaves were ground using a grinder and sieved through a 30-mesh screen. The powdered samples were then vacuum-sealed in polyethylene bags, which were subsequently placed in a desiccator at room temperature. All samples were preserved and used within 30 days.

Dried leaf powder (1.0 g) was extracted with ethanol : water mixtures at 30 : 70, 50 : 50, and 100 : 0 (v/v), using a 1 : 10 (w/v) sample-to-solvent ratio at room temperature. Extractions were performed three times (3 h×10 ml each) with the assistance of ultrasound (20 kHz, 100 W). After each cycle, the mixture was centrifuged, and the supernatants were combined and concentrated using a rotary evaporator (R-100, BUCHI Labortechnik AG, Switzerland) to obtain crude extracts. Extraction yield was expressed as the percentage of dry mass obtained after the extraction process and was calculated using equation (1), where m is the weight of the dried extract (g), and M is the weight of the initial dry plant powder (g). 
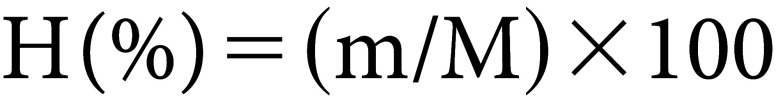
(1)

#### Assessment of total phenolic content, flavonoid content, rosmarinic acid, and antioxidant activity of *E. asperula*: Total phenolic content

The TPC was determined using the Folin–Ciocalteu reagent according to a modified method of Singleton et al. (1999). A stock solution of gallic acid (1000 µg ml^−1^) was prepared by dissolving 5 mg of anhydrous gallic acid (M=188.14 g mol^−1^) in ethanol to a final volume of 5 ml, and subsequently diluted to obtain standard solutions of 0, 25, 50, 100, 150, and 200 µg ml^−1^ for the calibration curve. For the assay, 500 µl of each standard solution (0, 20, 40, 80, and 100 µg ml^−1^) or appropriately diluted extract was transferred into Eppendorf tubes, followed by 2.5 ml of Folin–Ciocalteu reagent. After incubation in the dark for 4–5 min, 2.0 ml of 7.5% (w/v) Na_2_CO_3_ solution was added, and the mixtures were incubated at room temperature in the dark for 60 min. After incubation, 200 µl from each tube was transferred into a 96-well microplate. The absorbance was measured at 765 nm using a UV-Visible spectrophotometer (UV-Vis V730, Jasco, Japan). The absorbance-optical density (AOD) was calculated using equation (2). TPC was calculated from the calibration curve and expressed as milligrams of gallic acid equivalents (GAE) per gram of dry weight (DW). 
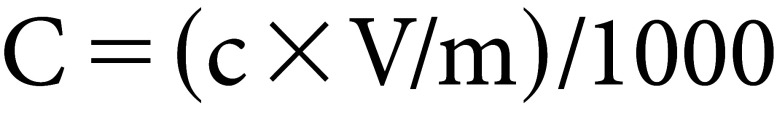
(2) Where: C is the TPC (mg gallic acid equivalents (GAE) per g dry weight), c is the concentration obtained from the calibration curve with gallic acid (µg ml^−1^), V is the volume of the extract solution used in the assay (ml), m is the weight of the dried extract present in volume (g).

#### Assessment of total phenolic content, flavonoid content, rosmarinic acid, and antioxidant activity of *E. asperula*: Flavonoids content

The flavonoid content in the samples was determined using an aluminium chloride colourimetric method based on forming a characteristic complex between aluminium chloride (AlCl_3_) and the functional groups of flavonoids (Zhishen et al. 1999). Methanol was used to dilute the extract to a concentration of 1.0 mg ml^−1^, and quercetin standard solutions were prepared at concentrations of 20, 40, 60, 80, and 100 µg ml^−1^. A 10% AlCl_3_ solution and a 1 M CH_3_COOK solution were diluted with distilled water for the assay.

For the assay, 0.5 ml of quercetin standard solution (at concentrations of 20, 40, 60, 80, and 100 µg ml^−1^) was mixed with 1.5 ml of methanol and allowed to react for 5 min. Then, 0.1 ml of 10% AlCl_3_ solution was added, and the mixture was incubated for 6 min. Subsequently, 0.1 ml of 1 M CH_3_COOK solution and 2.8 ml of distilled water were added. The mixture was shaken well and left to stand at room temperature for 45 min. After incubation, the absorbance was measured at 415 nm using a UV-Visible spectrophotometer. The TFC was calculated according to equation (3). 
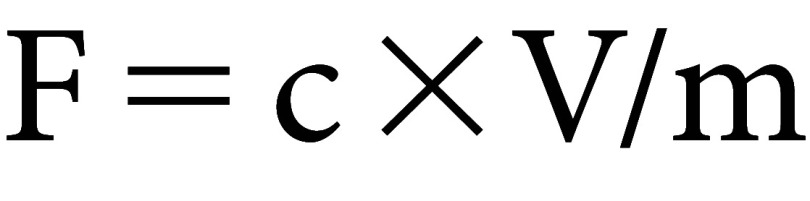
(3) Where: F is the total flavonoid content (mg quercetin equivalents (QE) per g dry weight), c is the concentration obtained from the quercetin calibration curve (µg ml^−1^), V is the volume of the extract solution used in the assay (ml), m is the weight of the dried extract present in volume V (g).

The results were expressed as milligrams of quercetin equivalents (mg QE) per gram of dry extract, allowing for comparison and evaluation of flavonoid content among different samples. The experiment was performed in triplicate. The optical density values were recorded and used to construct a calibration curve, which was then applied to determine the total flavonoid content in the extract samples.

#### Assessment of total phenolic content, flavonoid content, rosmarinic acid, and antioxidant activity of *E. asperula*: Rosmarinic acid

The RosA content in the *E. asperula* leaf extract was quantified using the UHPLC system following the Ştefănescu method (Ştefănescu 2025), with modifications suitable for *E. asperula* samples. This analysis was performed on an Acquity UPLC system (WATERS ACQUITY UPLC H-Class System, USA) using an ACQUITY™ Premier BEH-C18 column (1.7 µm, 2.1 mm×50 mm). The samples were injected through the column using the integrated pump system of the ACQUITY UPLC H-Class, operated under a high-gradient mode. The mobile phase consisted of water (A) containing 0.1% formic acid and acetonitrile (B) also containing 0.1% formic acid. The mobile phase flow rate was at 0.3 ml min^−1^, with the gradient of solvent B programmed as follows: 0 min - 10% B; 1.5 min - 70% B; 2.0 min - 70% B; 3.0 min - 10% B; and 5.0 min - 10% B. The total run time was 20 min, including the mobile phase equilibration period.

#### Determination of free radical scavenging assay

The antioxidant capacity of the extracts, based on their ability to scavenge DPPH radicals, was evaluated using the method reported by Wu et al. (2003).

The extracts were diluted in methanol to prepare a range of concentrations. Ascorbic acid was used as the positive control and diluted to 25, 20, 15, 10, and 5 µg ml^−1^ concentrations. To perform the DPPH assay, 100 µl of DPPH solution was mixed with 100 µl of each diluted extract or standard in individual microplate wells. The mixtures were incubated in the dark for 30 min, and absorbance was measured at 517 nm using a spectrophotometer. Methanol was used as the blank to replace the extract solution. The experiment was performed in triplicate.

A 96-well microplate was prepared by adding 100 µl of test sample or standard to each well, followed by 100 µl of 0.6 mM DPPH (2,2-diphenyl-1-picrylhydrazyl) solution. The mixtures were thoroughly mixed and incubated at 37°C for 30 min in the dark. After incubation, the optical density was measured at 517 nm using a Bio-Rad Benchmark Plus microplate reader (Bio-Rad, USA). Each concentration was tested in triplicate. The DPPH radical scavenging activity was calculated using equation (4). 
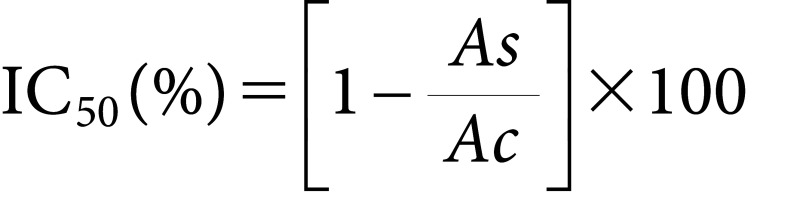
(4) Where Ac is the absorbance of the negative control and As is the absorbance of the sample. IC_50_ values were calculated as the concentration required to achieve 50% inhibition of DPPH radical activity.

Based on the linear relationship between DPPH radical scavenging activity and sample concentration, the concentration required to inhibit 50% of the DPPH radicals (IC_50_, µg ml^−1^) was determined. The IC_50_ value was calculated from the linear regression equation of the calibration curve: y=ax+b.

### Establishment of the micropropagation from the node explants of *E. asperula*

#### Evaluation of the impact of sterilization time on the viability and contamination rate of node explants

Node segments were excised from nursery-grown plants, with a 2.0–3.0 cm length. The explants were pre-cleaned using a diluted soap solution to remove surface dust and dirt. Subsequently, they were shaken in the soap solution for 5 min, rinsed thoroughly with water, and placed under running tap water for 30 to 60 min.

After surface cleaning, the explants were transferred to a laminar airflow cabinet for sterilization. They were treated with 70% ethanol for 30 s and rinsed with sterile distilled water. After that, the explants were sterilized in a 0.1% (w/v) HgCl_2_ solution for different durations (12, 16, and 20 min), followed by four rinses with sterile distilled water to remove disinfectant. After sterilization, necrotic tissues were removed, and the explants were trimmed into node segments of 1.0–1.5 cm in length. These node explants were cultured on MS medium without plant growth regulators. This experiment was conducted in three replicates, with each replicate consisting of 10 culture vessels, each containing one explant. After two weeks of culture, data were collected, such as the survival rate (%) and contamination rate (%) of the explants. 
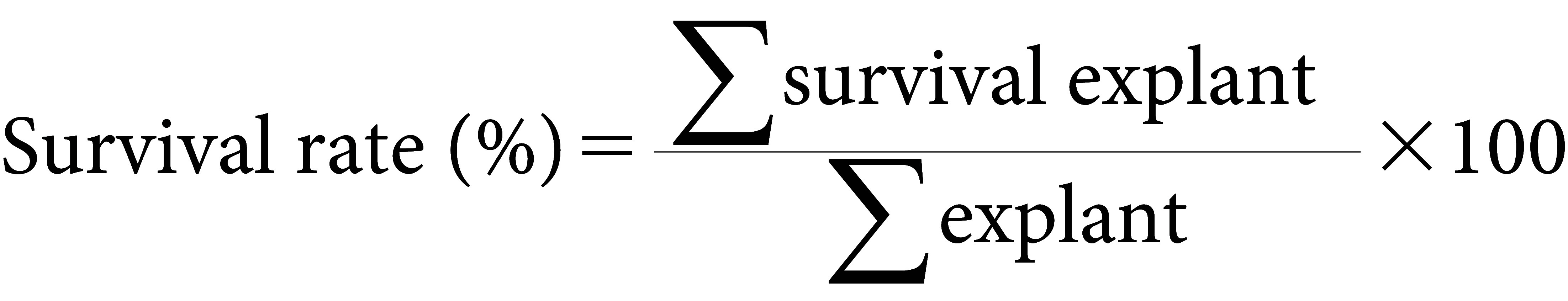

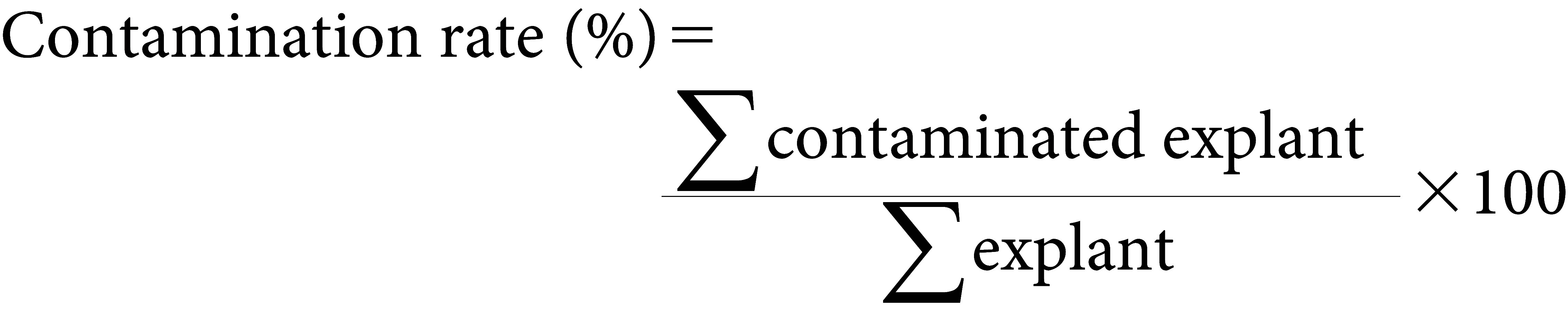


#### Evaluation of the impact of KIN on the shoot regeneration

The previous experiment’ surviving and uncontaminated node segments were subcultured onto MS medium supplemented with KIN at concentrations of 0.5, 1.0, 1.5, 2.0, 2.5, 3.0, 3.5, 4.0, 4.5, and 5.0 mg l^−1^. The treatment without KIN was used as the control. Each treatment consisted of 10 culture vessels (one explant per vessel) and was replicated three times. After four weeks of culture, data were collected based on the following parameters, including percentage of explants forming shoots (%), number of shoots per explant, and shoot height (cm).

#### Evaluation of the impact of BA and 2-iP on the shoot proliferation

In vitro *E. asperula* apical shoots of with a height of approximately 1.0 cm were cultured in 500 ml culture vessels containing 50 ml of MS medium supplemented with 30 g l^−1^ sucrose, 8 g l^−1^ agar, and either BA or 2-iP at various concentrations, such as 0.5, 1.0, 1.5, 2.0, 2.5, 3.0, 3.5, and 4.0 mg l^−1^. MS medium without BA was used as the control treatment. Each experiment was conducted in triplicate, with five vessels per replicate and three explants per vessel. After four weeks of culture, data were recorded for the following parameters: percentage of shoot formation (%), number of shoots per explant, shoot height (cm), and number of leaves per shoot.

#### Evaluation of the impact of NAA and IBA on the root formation

In vitro *E. asperula* apical shoots with a height of approximately 1.5 cm were cultured in a 500 ml flask containing 50 ml of MS medium supplemented with 30 g l^−1^ sucrose, 8 g l^−1^ agar, and either NAA or IBA at various concentrations, including 0.5, 1.0, 1.5, 2.0, 2.5, and 3.0 mg l^−1^. MS medium without auxin was used as the control treatment. Each experiment was conducted in triplicate, with three flasks per replicate, and three explants per flask. After four weeks of culture, data were recorded for the following parameters: percentage of root formation (%), number of roots per explant, and root length (cm).

#### Statistical analysis

Raw data were calculated and processed using Microsoft Excel 2016. One-way analysis of variance (ANOVA) was performed, followed by Tukey’s Honest Significant Difference (HSD) test for multiple comparisons at a significance level of *p*<0.05, using Stat Graphic software. The final results are presented as Mean±SD, where Mean represents the average value and SD indicates the standard deviation.

## Results and discussion

### Assessment of genetic diversity of *E. asperula*

The Internal Transcribed Spacer (ITS) region comprises non-coding sequences within the nuclear genome’s ribosomal DNA (rDNA) and is particularly prevalent among photosynthetic organisms. This region flanks the 5.8S ribosomal RNA gene, including the variable ITS1 and ITS2 sequences. It plays a crucial role in the production of rRNA, which is essential for ribosome biogenesis. The ITS regions are arranged in tandem within the rDNA, following the sequence of 18S, 5.8S, and 28S/26S rRNA genes. Specifically, ITS1 is located between the 18S and 5.8S genes, while ITS2 is positioned between the 5.8S and 28S genes (Mir et al. 2021). These coding regions are highly conserved, enabling the design of universal primers for PCR amplification. This facilitates taxonomic classification and phylogenetic reconstruction, particularly in identifying plant species. The ITS regions have demonstrated the ability to discriminate over 1,700 angiosperm species and have been successfully used to identify approximately 4,800 medicinal plants, achieving an accuracy rate of 90–93%. According to Lola et al. (2025), the ITS region was also utilized for species identification of *E. microphylla*, with phylogenetic analysis revealing a bootstrap value of 100% for *E. microphylla* in their study. Similarly, a previous study by Nguyen et al. (2017) also confirmed the role of ITS regions in assessing the genetic diversity of *E. asperula* originating from Hoa Binh Province, Vietnam. These findings further support the high reliability of the ITS sequence in distinguishing species within the genus *Ehretia*, and demonstrate its suitability for the species identification of *E. asperula*.

**Figure 1:**
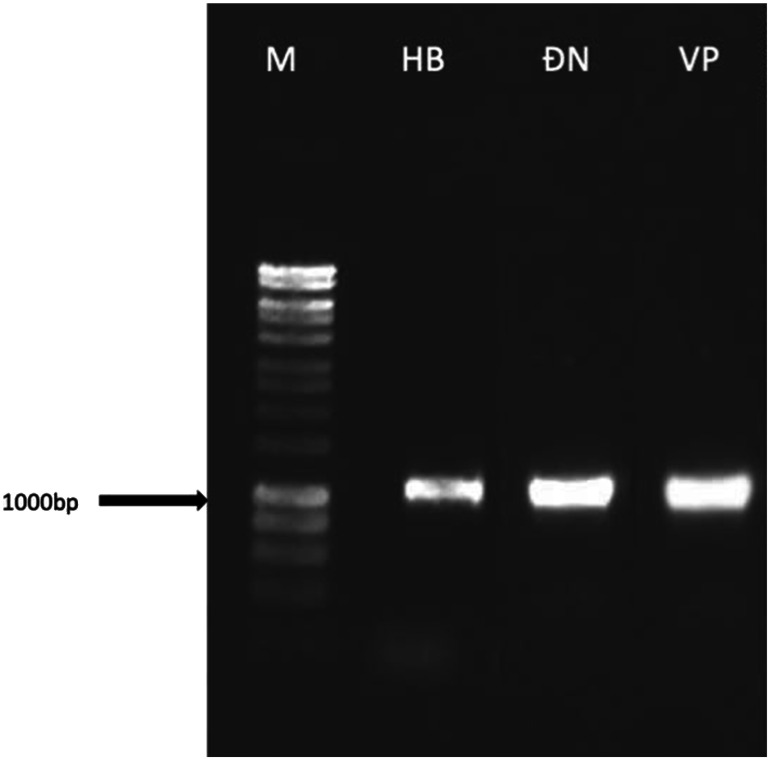
Figure 1. PCR products using the ITS primer pair from *E. asperula* collected from three different regions, including Hoa Binh (HB), Dong Nai (ĐN), and Vinh Phuc (VP). M, DNA marker.

The PCR results showed clear and bright DNA marker bands without any smearing, indicating efficient PCR amplification and absence of DNA fragmentation (Figure 1). The nucleotide sequences obtained after decoding were compared with the GenBank database of the U.S. National Center for Biotechnology Information (NCBI). Phylogenetic trees were constructed using MEGA X software with a bootstrap value calculated from 1000 replicates. The ITS sequences of *E. asperula* originating from three different regions were submitted to GenBank under the following accession IDs: PQ237099 (Hoa Binh); PV973038 (Dong Nai); PV973039 (Vinh Phuc).

**Figure 2:**
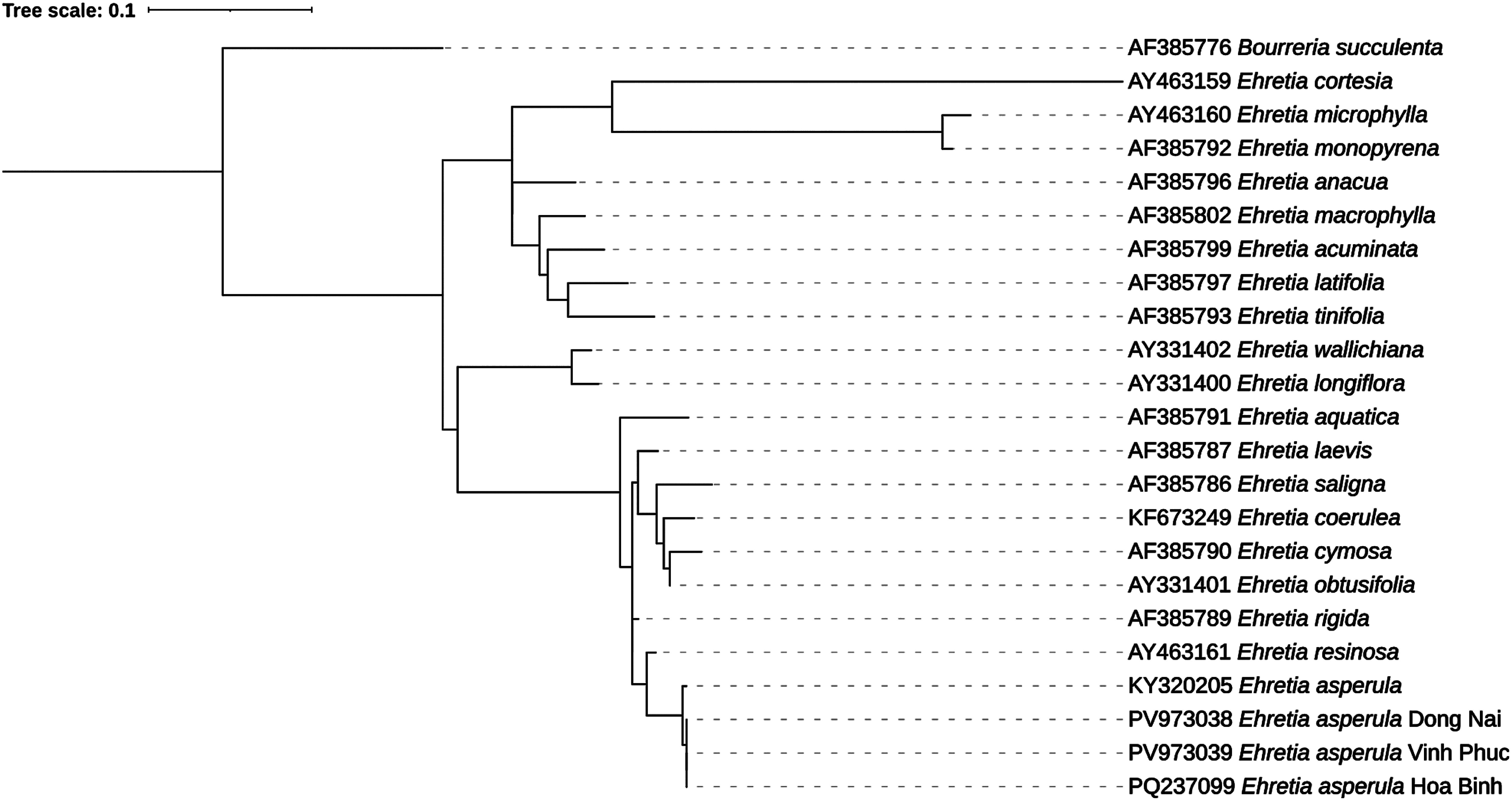
Figure 2. Phylogenetic tree derived from maximum likelihood analysis of the ITS region of 23 sequences. *Ehretia asperula* Dong Nai (PV973038), *Ehretia asperula* Vinh Phuc (PV973039), *Ehretia asperula* Hoa Binh (PQ237099) represent the samples analyzed in this study.

The phylogenetic tree constructed from the ITS sequence region delineates the species within the genus *Ehretia*, reflecting their evolutionary relationships. In this phylogeny, *E. asperula* were placed in a distinct clade with high similarity, indicating the species’ genetic distinctiveness and stability compared to other species within the genus (Figure 2). At the terminal branch of the phylogenetic tree, three *E. asperula* plants originating from Dong Nai, Vinh Phuc, and Hoa Binh provinces formed a well-supported monophyletic clade with a bootstrap value of 100%, indicating ITS sequence similarity among populations from different geographic regions. *E. asperula* (KY320205) was also grouped within this clade, forming a clearly defined *E. asperula* cluster supported by strong bootstrap values. These findings align with those of Nguyen et al. (2017), who, based on analyses of the ITS1 and matK regions, observed low genetic differentiation among *E. asperula* species and identified a monophyletic clade supported by bootstrap values exceeding 92%.

Species such as *E. rigida* (AF385789), *E. resinosa* (AY463161), and *E. saligna* (AF385786) formed distinct clades with bootstrap values ranging from 90% to 100%. This indicates that these species possess sufficiently divergent ITS characteristics for clear delimitation while reflecting close evolutionary relationships. The clade containing *E. laevis* and *E. aquatica* exhibited a bootstrap value of 100%, supporting the hypothesis that these species share a close common origin and may belong to the same subclade. Additionally, the clade comprising *E. wallichiana* (AY331402) and *E. longiflora* (AY331400) was distinctly separated with 100% bootstrap support. Similarly, *E. cymosa* (AF385790) and *E. saligna* (AF385786) showed high bootstrap values, representing independent evolutionary units.

At the basal part of the phylogenetic tree, species such as *E. microphylla*, *E. cortesia*, and *E. monopyrena* formed a more distantly related clade, separated from other branches with a bootstrap support of 97%. Finally, *Bourreria succulenta* (AF385776) was appropriately used as the outgroup, belonging to a different genus within the *Boraginaceae* family. This choice helps to root the phylogenetic tree and ensures an objective analysis of the evolutionary relationships within the genus *Ehretia*. The group comprising *E. tinifolia*, *E. latifolia*, and *E. acuminata* showed moderate bootstrap support (78–79%), indicating that this relationship is relatively reliable.

### Assessment of total phenolic content, flavonoid content, rosmarinic acid concentration, and antioxidant activity of *E. asperula*

#### Total phenolic content

**Figure 3:**
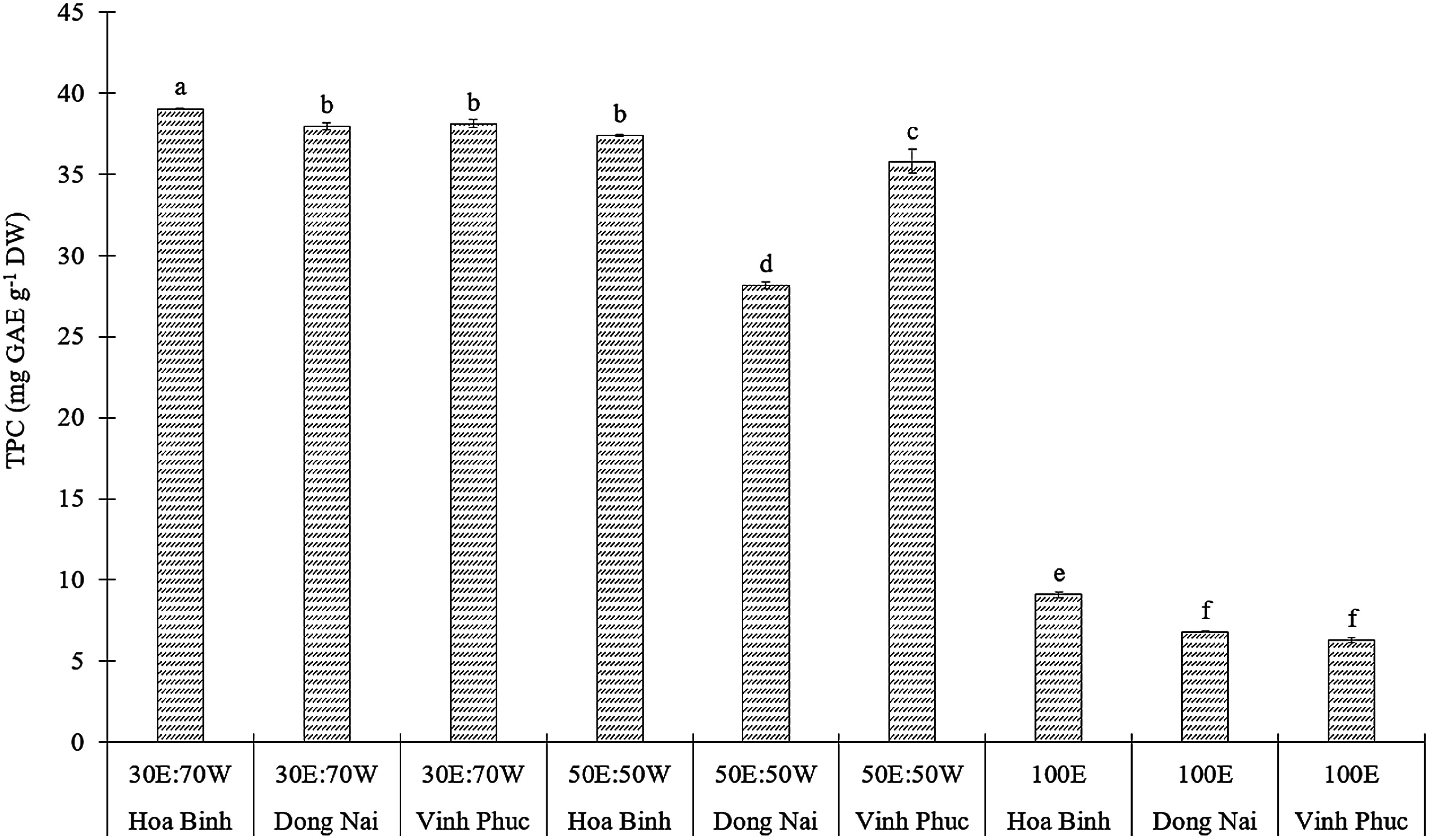
Figure 3. The TPC of *E. asperula* leaves from different sources was extracted using solvents with varying ethanol-to-water ratios, including 30% ethanol : 70% water (30E : 70W), 50% ethanol : 50% water (50E : 50W), and 100% ethanol (100E). Means followed by different letters indicate significant differences according to Tukey’s HSD test (*p*<0.05).

The results revealed that the TPC in *E. asperula* leaves varied significantly depending on the geographical origin and the ratio of ethanol and water in the extraction solvent (*p*<0.05, Figure 3). Among the tested solvents, 30% ethanol and 70% water (v/v) yielded the highest TPC, with the Hoa Binh sample exhibiting a peak value of 39.03±0.03 mg GAE g^−1^ DW. A gradual decline in TPC was observed with solvent ratios of 50 : 50 (v/v) and 100% ethanol. The Vinh Phuc sample extracted with absolute ethanol contained the lowest TPC, reaching only 6.28 mg GAE g^−1^ DW. Recent studies on *E. asperula* have demonstrated that under optimized extraction conditions using Response Surface Methodology (RSM), a solvent system containing 60% ethanol combined with ultrasound-assisted extraction for 20 min had a TPC of up to 94.2±1.4 mg GAE g^−1^ DW (Duc et al. 2024). In contrast, Le et al. (2024) reported that conventional extraction without ultrasonic assistance, using ethanol : water (70 : 30, v/v), yielded a TPC of 55 mg GAE g^−1^ DW.

In the study by Nguyen et al. (2024) on *E. acuminata*, ethanolic extraction (70%) from fruit at room temperature had a TPC of 118.76±2.20 mg GAE g^−1^ DW, which was markedly higher compared to several other congeneric species and extraction protocols. In contrast, extracts from *E. cymosa* leaves obtained using 80% methanol over 24 h at room temperature resulted in a TPC of 65.33±1.40 mg GAE g^−1^ DW (Ashagrie et al. 2023). These showed the variation in phenolic content across species, plant organs, and solvent systems.

Current findings and available studies suggest that members of the genus *Ehretia*, particularly *E. asperula* and *E. acuminata*, possess a high biosynthetic capacity for phenolic compounds. The use of 30% ethanol - 70% water solvent, especially when combined with extraction-enhancing techniques such as ultrasonication, has been shown to significantly improve the recovery of these compounds compared to extraction using either water or absolute ethanol alone.

#### Total flavonoids content

**Figure 4:**
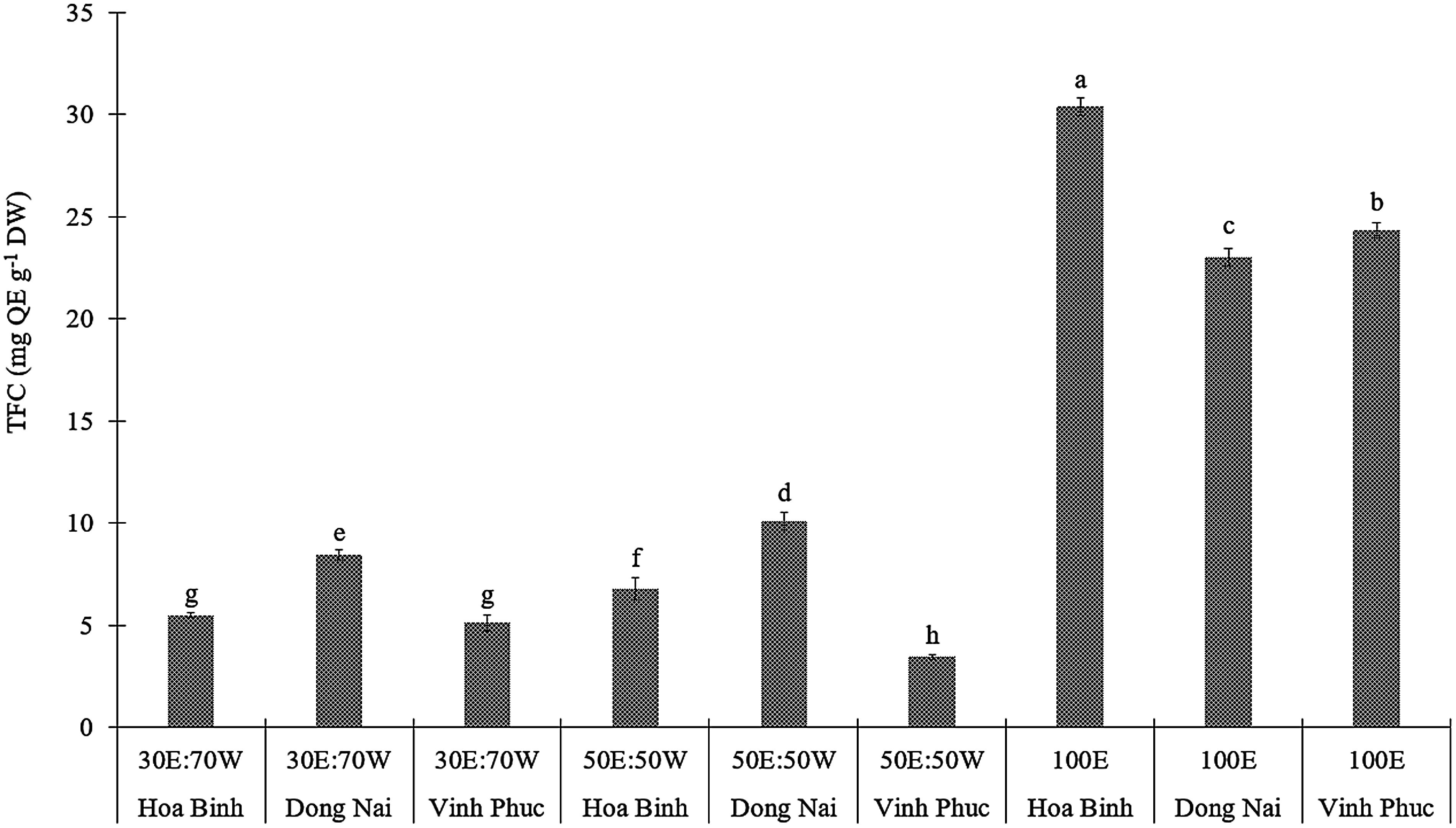
Figure 4. The TFC of *E. asperula* leaves from different sources was extracted using solvents with varying ethanol-to-water ratios, including 30% ethanol : 70% water (30E : 70W), 50% ethanol : 50% water (50E : 50W), and 100% ethanol (100E). Means followed by different letters indicate significant differences according to Tukey’s HSD test (*p*<0.05).

The results presented in the table indicated that the TFC in extracts of *E. asperula* leaves, determined using the aluminium chloride complexation method, varied significantly depending on both the solvent ratio and the geographical origin of the samples (*p*<0.05, Figure 4). When combined with ultrasonic-assisted extraction, absolute ethanol yielded the highest TFC, particularly in the Hoa Binh sample, which reached 30.3874 mg QE g^−1^ DW. In contrast, solvent mixtures of 30% ethanol and 70% water and 50% ethanol and 50% water resulted in significantly lower TFC values, ranging from 3.45 to 10.08 mg QE g^−1^ DW. These findings showed the solvent polarity’s crucial role in flavonoid recovery efficiency. Absolute ethanol is more effective at dissolving non-polar or low-polarity flavonoid compounds, whereas the presence of water increases overall solvent polarity, thereby reducing the extraction efficiency of such compounds. Recent studies have reported similar findings in other species within the *Boraginaceae* family. In the study by Ashagrie et al. (2023), ultrasonic-assisted extraction of *E. cymosa* using 80% methanol at approximately 40°C for 30 min yielded the highest TFC, reaching 48.20±1.12 mg QE g^−1^ DW.

A study on *Borago officinalis*, a species within the *Boraginaceae* family, reported a flavonoid content of 7.80±0.41 mg QE g^−1^ DW when extracted using methanol combined with ultrasonic-assisted extraction for 20 min (Michalak et al. 2023). The results of this study indicated that the flavonoid content in *E. asperula* samples originating from the Dong Nai is comparable but slightly higher than that of *Borago officinalis* leaf extract obtained under similar extraction conditions. Similarly, in the study of *Echium italicum*, another species within the *Boraginaceae* family, the authors employed an optimized ultrasound-assisted extraction method, which had TFC ranging from 4.5 to 7.2 mg QE g^−1^ DW depending on the extraction temperature and solvent system (Gheisary et al. 2025). These results are comparable to those obtained from *E. asperula* leaves samples from Hoa Binh and Vinh Phuc.

A comprehensive study on various *Boraginaceae* species from Macedonia reported TFC ranging from 3.8 to 6.9 mg QE g^−1^ DW, depending on the species and extraction conditions (Petreska Stanoeva et al. 2023). Compared to other *Boraginaceae* members, the TFC in *E. asperula* leaf extracted with a 30% ethanol and 70% water solvent combined with ultrasonication was relatively high, with the Dong Nai sample showing significantly higher values than the other treatments.

#### Rosmarinic acid

**Figure 5:**
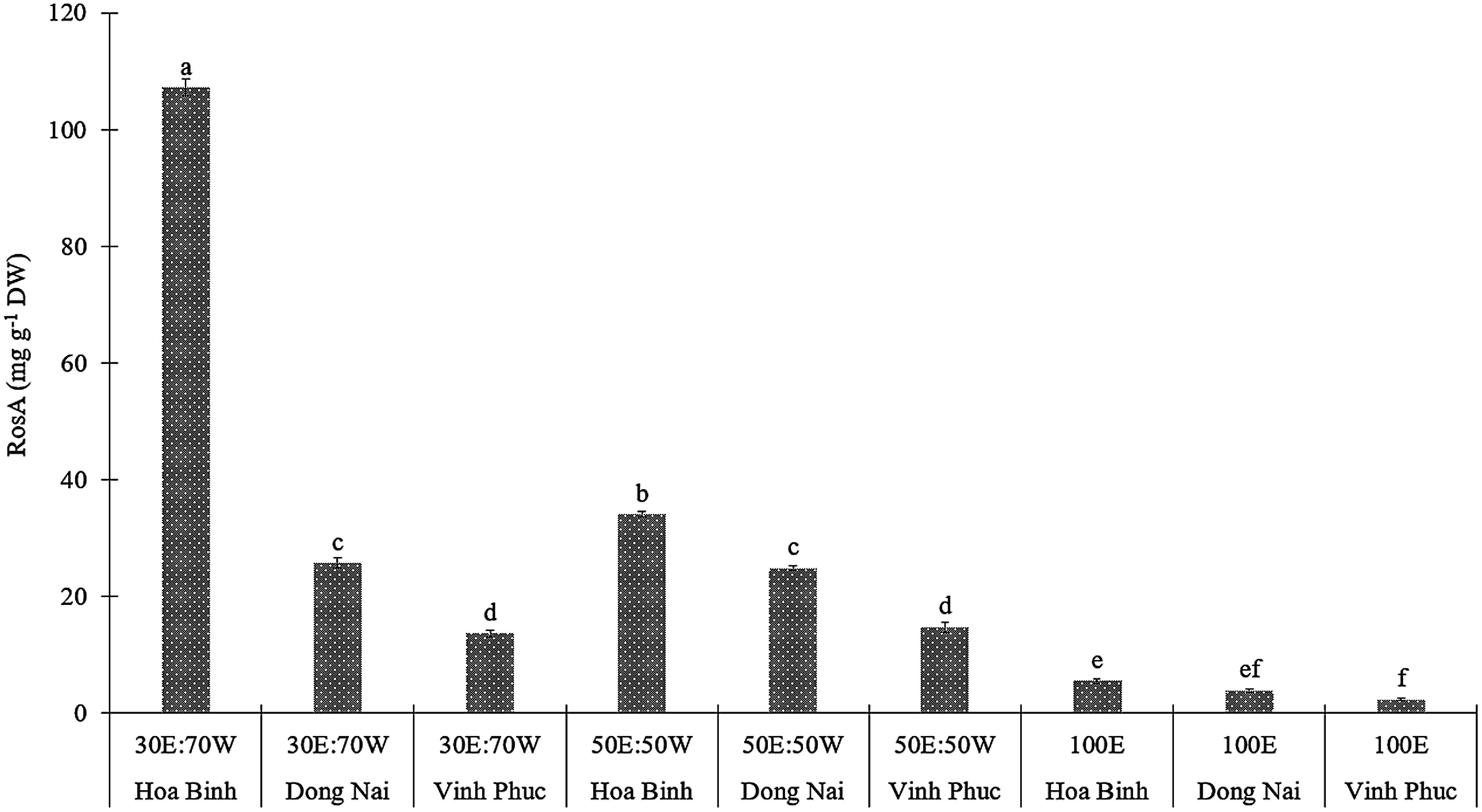
Figure 5. The RosA content of *E. asperula* leaves from different sources was extracted using solvents with varying ethanol-to-water ratios, including 30% ethanol : 70% water (30E : 70W), 50% ethanol : 50% water (50E : 50W), and 100% ethanol (100E). Means followed by different letters indicate significant differences according to Tukey’s HSD test (*p*<0.05).

Similar to the abovementioned results, the RosA content in *E. asperula* leaves was significantly influenced by the ethanol/water solvent ratio and the geographical origin (*p*<0.05, Figure 5). When extracted with 30% ethanol and 70% water, the Hoa Binh sample exhibited the highest RosA content (107.153 mg g^−1^ DW). This value was approximately four times higher than the Dong Nai sample and nearly eight times higher than the Vinh Phuc sample under the same extraction conditions. The solvent combination of ethanol and water at a 30 : 70 ratio yielded the highest extraction efficiency across all tested sample origins, indicating its suitability for dissolving and releasing polar compounds such as RosA. Rosmarinic acid is a polyphenolic compound characterized by two aromatic rings bearing multiple hydroxyl (-OH) and carboxylic (-COOH) groups, which confer high polarity and hydrophilicity to the molecule. When using absolute ethanol as the extraction solvent (100% ethanol), the low polarity of the solvent system limits the solubility of RosA due to insufficient polarity in the extraction environment. In contrast, the inclusion of water in the 30% ethanol and 70% water solvent system increases the overall polarity of the medium. It facilitates the swelling of plant cell walls, thereby enhancing the diffusion of RosA into the solvent. This observation is consistent with the findings reported by Sik et al. (2019). Using water as a sole solvent often leads to the co-extraction of undesirable impurities such as sugars and mineral salts, which can interfere with subsequent purification and quantification processes. Therefore, a mixed ethanol-water solvent is optimal, as it balances selective solubility and extraction efficiency. Specifically, the 30% ethanol and 70% water solvent ratio may promote optimal energy distribution during ultrasonic treatment, enhancing solvent permeability into plant tissues and improving overall extraction yield.

Previous studies have also reported the effectiveness of combined solvent systems and ultrasonic-assisted extraction in enhancing the recovery of RosA from other plant species. Specifically, the study by Duc et al. (2024) demonstrated that the combination of ultrasound-assisted extraction and an ethanol-water solvent system effectively optimized the recovery of RosA from *E. asperula*, with the optimal extraction parameters being largely consistent with those observed in the present study. In addition, Gautam et al. (2024) reported that ultrasonic-assisted extraction not only shortens extraction time and reduces the required temperature but also enhances the release of compounds such as RosA and hydroxycaffeic acid from medicinal plants belonging to the *Boraginaceae* family. In the study by Istifli (2021), extraction of *Onosma bourgaei* leaves, a species in the *Boraginaceae* family, using 70% ethanol combined with ultrasonic assistance yielded a RosA content of approximately 21.5 mg g^−1^ DW (quantified by LC-MS/MS). This value is lower than that obtained from *E. asperula* leaves collected from Hoa Binh (107.153 mg g^−1^) in the present study. These findings reflect interspecific biological differences and the enhanced efficiency of the optimized solvent system.

Sik et al. (2019) provided a comprehensive overview of analytical methodologies for the quantification of RosA, including high-performance liquid chromatography (HPLC), liquid chromatography-mass spectrometry (LC-MS), and ultraviolet-visible (UV-Vis) spectrophotometry. Among these techniques, LC-MS/MS has been recognized as the most accurate and selective method, owing to its ability to simultaneously determine target compounds’ molecular weight and specific fragmentation patterns. In particular, the LC-MS/MS method employing acetonitrile and formic acid as the mobile phase on a C18 reversed-phase column has been established as the method of choice for analyzing structurally complex phenolic acids in plant-derived matrices.

While solvent type clearly influenced RosA extraction efficiency, the comparatively higher yields in the Hoa Binh samples suggest that additional factors may also contribute. Possible influences include environmental conditions (soil composition, altitude, light intensity…) as well as subtle genetic variation not captured by ITS analysis. These observations indicate that both extrinsic and intrinsic factors may play a role in shaping RosA accumulation.

### Determination of free radical scavenging assay

**Figure 6:**
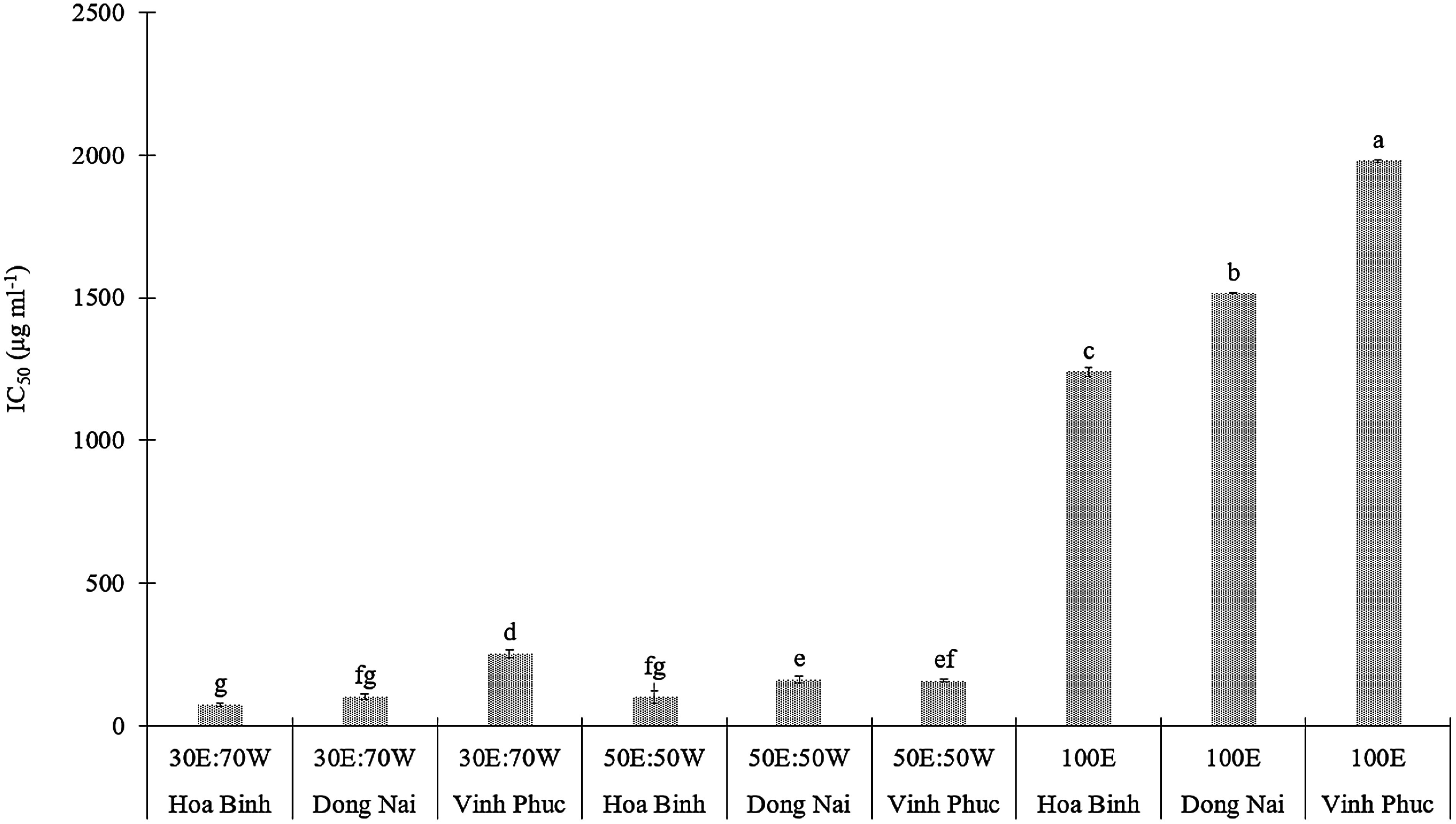
Figure 6. Effect of sample origin and solvent type on the antioxidant activity of *E. asperula* leaves from different sources. Means followed by different letters indicate significant differences according to Tukey’s HSD test (*p*<0.05). Treatments were coded according to solvent composition as follows: 30% ethanol : 70% water (30E : 70W), 50% ethanol : 50% water (50E : 50W), and 100% ethanol (100E).

Consistent with previous experiments, the results indicate that both the extraction solvent and the geographical origin of *E. asperula* samples significantly influence the antioxidant activity of this species (*p*<0.05, Figure 6). Under extraction with 30% ethanol and 70% water (v/v), the Hoa Binh sample exhibited the most potent antioxidant activity, with an IC_50_ value of 75.03±5.18 µg ml^−1^, followed by the samples from Dong Nai (IC_50_=102.93±10.69 µg ml^−1^) and Vinh Phuc (IC_50_=253.66±12.88 µg ml^−1^). When the ethanol concentration was increased to a 50 : 50 ratio of ethanol and water, the antioxidant activity significantly decreased, with IC_50_ values ranging from 103.35 to 163.21 µg ml^−1^. Notably, when pure ethanol was used as the solvent, the IC_50_ value increased sharply, exceeding 1200 µg ml^−1^ and reaching nearly 2000 µg ml^−1^. These results indicate a significant decline in the extraction efficiency of antioxidant compounds under anhydrous conditions, highlighting the essential role of water in the solvent mixture for effective extraction of bioactive substances. These findings support the hypothesis that water in the extraction solvent facilitates the extraction of highly polar phenolic and flavonoid compounds, which are the main bioactive agents involved in free radical scavenging. This effect has been demonstrated in a study on *E. acuminata*, where an ethanol : water mixture at an optimal ratio of 30 : 70 yielded the lowest IC_50_ value of 24.83 µg ml^−1^, which is comparable to the antioxidant potency of the ascorbic acid standard (Nguyen et al. 2024).

Based on linear regression analysis, the correlation between the secondary metabolites content and antioxidant activity showed that TPC and TFC showed statistically significant correlations with antioxidant activity, as indicated by coefficients of determination of R^2^=91.32% (*p*=0.0001<0.05) and R^2^=79.86% (*p*=0.0012<0.05), respectively (Table 2). In contrast, the content of RosA did not show a statistically significant correlation with DPPH radical scavenging activity (R^2^=28.38%; *p*=0.1397>0.05). This suggests that RosA may not be the principal compound contributing to the antioxidant activity in the *E. asperula* samples analyzed. Similarly, extracts from *Orthosiphon grandiflorus*, which are known to contain high levels of RosA, exhibit IC_50_ values ranging from approximately 80 to 100 µg ml^−1^, comparable to the Dong Nai sample in the present study (Nuengchamnong et al. 2011).

**Table 2:** Table 2. Simple linear regression between inhibition capacity and total phenolic content, total flavonoid content, and rosmarinic acid content.

Regression	IC vs TPC	IC vs TFC	IC vs RosA
R suqared	91.3197	79.856	28.3832
Linear model	IC=1901.98−483194*TPC	IC=−238.192+66.0837*TFC	IC=936.535+12.2262*RosA
*p* value	0.0001	0.0012	0.1397

Among the analyzed samples, the leaf extract of *E. asperula* originating from Hoa Binh demonstrated the most potent antioxidant activity, underscoring its promise as a natural antioxidant source. This particular sample contained 107.153 mg RosA g^−1^ DW and exhibited an IC_50_ value of 75.03 µg ml^−1^. Comparable antioxidant activity has been reported for *Rosmarinus officinalis* L., a well-known antioxidant-rich species that accumulates polyphenolic compounds, particularly RosA, as major secondary metabolites. Extracts of *R. officinalis* have been shown to exhibit strong DPPH radical-scavenging activity, with IC_50_ values of approximately 130 µg ml^−1^, supporting the high antioxidant potential observed in the present study (Dhouibi et al. 2023). Recent studies have revealed that several species within the *Ehretia* genus contain RosA and are closely associated with various biological activities, including free radical scavenging, glycemic regulation, and anti-inflammatory effects (Guan et al. 2022). Among these, including our current investigation, *E. asperula* not only exhibits a high RosA content but also demonstrates notably vigorous antioxidant activity.

### Establishment of the process of the micropropagation of *E. asperula*

Despite the absence of genetic variation among *E. asperula* samples from the three different geographical origins based on ITS sequence analysis, notable differences were observed in their secondary metabolite profiles. The plants derived from Hoa Binh displayed the highest concentrations of these compounds, indicating their potential as the most suitable source material for micropropagation.

#### Evaluation of the impact of sterilization time on the viability and contamination rate of node explants

Surface sterilization is one of the critical steps in plant tissue culture, ensuring that explants are free from microbial contamination (Ankita and Handique 2010). The primary objective of this process is to eliminate microorganisms present on the surface of the explants, thereby maintaining aseptic conditions for healthy plant cell development. During the tissue culture process, contamination of explants can arise from various sources, with the primary cause often originating from the explant itself. Microorganisms such as fungi and bacteria commonly adhere to the surface of plant tissues, especially at wounds, stomata, or lenticels (Agrios 2005). Additionally, the nutrient-rich culture medium provides a favorable environment for microbial growth. In nutrient-rich media, microorganisms can proliferate more rapidly than plant tissues and may release toxic compounds into the medium, inhibiting cultured tissue growth and development (Razdan 2002). Therefore, explants must be sterilized using chemical disinfectants. These agents inhibit or eliminate microbial growth, and their concentrations must be carefully selected to ensure effective sterilization while minimizing toxicity to plant tissues (Razdan 2002). To sterilize explants, highly effective disinfectant solutions such as calcium hypochlorite (Ca(OCl)_2_), sodium hypochlorite (NaOCl), and mercuric chloride (HgCl_2_) are commonly used. The primary mechanism of action of these agents involves disrupting microbial cell membranes, inhibiting metabolic processes, and denaturing proteins (Liu et al. 2006). The success of sterilization depends on both the duration of exposure and the concentration of the disinfectants used.

**Figure 7:**
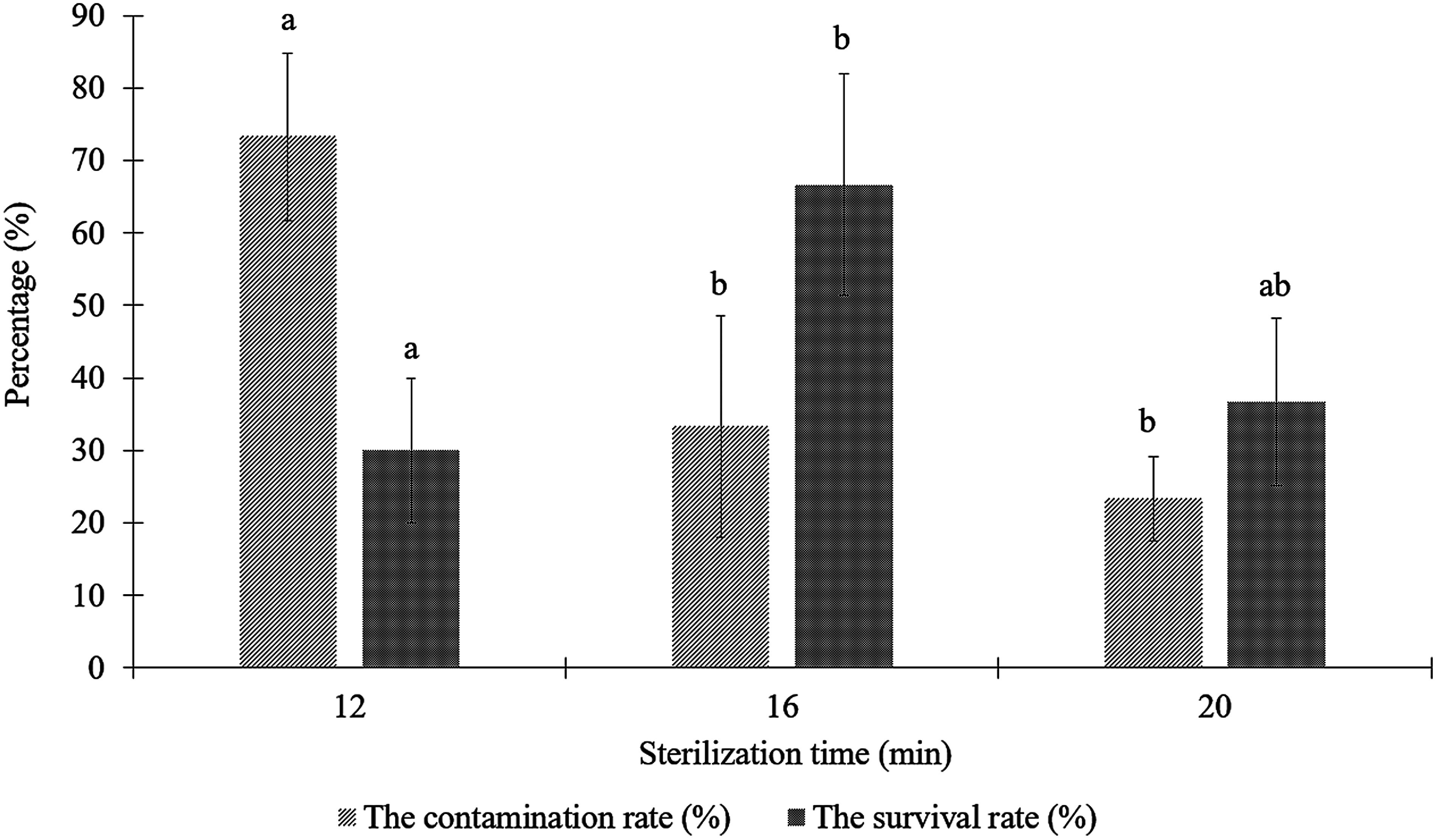
Figure 7. Effect of 0.1% HgCl_2_ on the sterilization time, the contamination rate and the survival rate of *E. asperula* node explants. Means followed by different letters indicate significant differences according to Tukey’s HSD test (*p*<0.05).

According to Liu et al. (2006), HgCl_2_ is a potent disinfectant that is highly toxic to microbial cells. It can destroy bacterial and fungal spores by disrupting SH bonds in protein molecules, leading to rapid microbial cell death when applied at appropriate concentrations and exposure times. These findings are consistent with the results of the present experiment, in which aseptic survival was observed across all tested treatments. In this experiment, the disinfectant concentration was fixed at 0.1% HgCl_2_, so the aseptic survival of *E. asperula* stem segments depended primarily on the duration of exposure. A 16-min treatment provided the most effective balance, with 66.67% survival and 33.33% contamination (Figure 7), while shorter exposure led to excessive contamination and longer exposure reduced contamination but severely compromised explant survival due to tissue damage. In this experiment, the relatively large SD observed at some time points likely reflects the limited sample size, where even minor variation in explant responses could substantially influence the calculated percentages.

#### Effect of KIN on the *E. asperula* shoot regeneration

Kinetin (KIN), a type of cytokinin, promotes cell division and is commonly added to in vitro culture media to enhance shoot regeneration rates (Davies 1995). After 4 weeks of culture, the recorded data indicated that KIN positively affected on shoot formation in *E. asperula* under in vitro conditions. The shoot induction rate of explants cultured on MS medium supplemented with KIN reached 100%, significantly higher than the control treatment (10.33%) (Table 3, Figure 10A). The number of shoots formed (1.0–1.67 shoots/explant), shoot height (1.0–2.1 cm), and number of leaves (1.57–5.30 leaves/shoot) increased proportionally with the concentration of KIN (0.5–3.0 mg l^−1^) added to the culture medium. Explants cultured on MS medium supplemented with 3.0 mg l^−1^ KIN produced the highest number of shoots (1.67 shoots/explant), which was statistically different (*p*<0.05) from the other KIN concentrations tested. Shoot proliferation in this treatment also performed best, with an average shoot height of 2.1 cm and 5.3 leaves per shoot. Similar results were reported by Tram et al. (2018), in which the supplementation of 3.0 mg l^−1^ KIN yielded the best shoot formation from *E. asperula* node segments, with an average of 1.57 shoots per explant and a mean shoot height of 1.37 cm. Adding exogenous cytokinin to the culture medium alters the levels of endogenous plant growth regulators within the explant and establishes a new gradient of endogenous hormone concentrations. This shift helps break dormancy and stimulates shoot induction. However, when higher concentrations of KIN (3.5–5.0 mg l^−1^) were used, these parameters decreased. Histological analysis (Figure 10B) revealed that node segments of *E. asperula* cultured on KIN-supplemented medium exhibited activation of lateral meristematic tissues, which gave rise to shoot primordia. These primordia further differentiated and elongated into shoots. In this experiment, some treatments showed larger standard deviations due to high variability among explants’ responses, which is common in shoot regeneration from ex vitro node culture experiments with a limited sample size. Increasing the number of replicates in future studies may help reduce variability and provide more robust statistical estimates for percentage data.

**Table 3:** Table 3. Effect of KIN on the *E. asperula* shoot regeneration from node explants after 4 weeks of culture.

KIN (mg l^−1^)	Percentage of shoot regeneration (%)	Number of shoots/explant	Shoot height (cm)	Number of leaves/shoot
Control	10.33^b^	1.00±0.00^d^	0.71±0.82^d^	0.93±0.20^e^
0.5	100.00^a^	1.00±0.00^d^	1.00±0.81^cd^	1.57±0.30^de^
1.0	100.00^a^	1.10±0.30^cd^	1.05±0.92^c^	1.63±0.29^cde^
1.5	100.00^a^	1.23±0.43^bcd^	1.13±1.09^c^	1.67±0.21^cde^
2.0	100.00^a^	1.32±0.47^bc^	1.22±0.98^bc^	1.71±0.36^bcde^
2.5	100.00^a^	1.40±0.49^ab^	1.45±0.91^b^	2.30±0.60^bcd^
3.0	100.00^a^	1.67±0.47^a^	2.10±1.31^a^	5.30±0.54^a^
3.5	100.00^a^	1.43±0.50^ab^	2.08±1.50^a^	2.53±0.53^b^
4.0	100.00^a^	1.23±0.43^bcd^	1.54±0.90^b^	2.47±0.27^bc^
4.5	100.00^a^	1.00±0.00^d^	1.13±1.09^c^	2.33±0.30^bcd^
5.0	100.00^a^	1.00±0.00^d^	1.09±0.89^c^	2.03±0.33^bcd^

Means followed by different letters differ significantly according to Tukey’s HSD test (*p*<0.05).

#### Effect of BA on the *E. asperula* shoot proliferation

After 4 weeks of culture, the number of shoots in the treatment using BA (1.67–6.27 shoots per explant) was higher than that in the BA-free treatment (1.20 shoots per explant) (Table 4). In treatments with BA concentrations from 1.0 to 3.0 mg l^−1^, the number of shoots increased proportionally with the BA concentration in the culture medium. However, the number of shoots decreased at higher BA concentrations (3.5–4.0 mg l^−1^). The highest number of shoots was obtained in the treatment with 3.0 mg l^−1^ BA, reaching 6.27 shoots per explant, with an average shoot height of 1.89 cm and 7.40 leaves per explant. This result is also consistent with the findings of Behera et al. (2015), who observed that in vitro shoots of *Bacopa monnieri* (L.) exhibited optimal growth when cultured on medium supplemented with 3.0 mg l^−1^ BA.

**Table 4:** Table 4. Effect of BA on the in vitro *E. asperula* shoot proliferation after 4 weeks of culture.

BA (mg l^−1^)	Number of shoots/explant	Shoot height (cm)	Number of leaves/shoot
Control	1.20±0.41^f^	1.43±0.19^e^	2.86±0.83^d^
0.5	1.67±0.61^ef^	1.48±0.25^de^	5.40±0.73^c^
1.0	2.07±0.79^e^	1.54±0.16^cde^	5.87±0.83^bc^
1.5	3.47±0.91^d^	1.68±0.17^bcd^	6.13±0.83^bc^
2.0	3.93±0.70^cd^	1.74±0.21^abc^	6.47±0.74^ab^
2.5	4.40±0.91^bc^	1.78±0.15^ab^	6.53±0.83^ab^
3.0	6.27±0.70^a^	1.89±0.13^a^	7.40±0.73^a^
3.5	5.07±0.59^b^	1.77±0.13^ab^	6.53±0.64^ab^
4.0	3.87±0.74^cd^	1.72±0.13^abc^	6.20±0.77^bc^

Means followed by different letters differ significantly according to Tukey’s HSD test (*p*<0.05).

#### Effect of 2-iP on the in vitro *E. asperula* shoot proliferation

In addition to BA, 2-isopentenyladenine (2-iP) is a naturally occurring cytokinin widely used for its ability to promote cell division and influence organ differentiation, particularly shoot differentiation (Adrian et al. 2003). In this experiment, in vitro *E. asperula* shoots were cultured on MS medium supplemented with various concentrations of 2-iP to evaluate its effects on shoot proliferation. The results showed statistically significant differences in all observed parameters among the 2-iP treatments, as well as the control after 4 weeks of culture (*p*<0.5, Table 5). Among the treatments, shoot explants cultured on MS medium supplemented with 1.5 mg l^−1^ 2-iP exhibited the highest values for all parameters, including the number of shoots (3.20 shoots per explant), shoot height (3.56 cm), and number of leaves per shoot (4.33 leaves). This was followed by the treatment with 2.0 mg l^−1^ 2-iP, with the number of shoots (2.10 shoots per explant), shoot height (1.98 cm) and number of leaves per shoot (3.67 leaves).

**Table 5:** Table 5. Effect of 2-iP on the in vitro *E. asperula* shoot proliferation after 4 weeks of culture.

2-iP (mg l^−1^)	Number of shoots/explant	Shoot height (cm)	Number of leaves/shoot
Control	1.20±0.41^c^	1.80±0.30^b^	2.87±0.83^b^
0.5	1.27±0.45^c^	1.98±0.36^b^	3.07±1.10^b^
1.0	1.27±0.45^c^	1.92±0.64^b^	3.40±0.98^ab^
1.5	3.20±0.86^a^	3.56±0.84^a^	4.33±0.97^a^
2.0	2.10±0.91^b^	1.98±0.45^b^	3.67±1.04^ab^
2.5	1.80±0.86^bc^	1.92±0.43^b^	3.27±1.28^ab^
3.0	1.67±0.72^bc^	1.65±0.32^b^	3.20±1.01^ab^
3.5	1.47±0.83^b^	1.61±0.56^b^	3.13±1.06^b^
4.0	1.20±0.41^c^	1.55±0.25^b^	2.60±0.98^b^

Means followed by different letters differ significantly according to Tukey’s HSD test (*p*<0.05).

In this experiment, BA stimulated shoot formation of the explants but was not effective in promoting shoot elongation. All explants cultured on medium supplemented with 3.0 mg l^−1^ BA showed a lower average shoot height than those cultured on medium with 1.5 mg l^−1^ 2-iP in the most effective treatment (Figure 10D, E). This can be explained by the presence of BA inhibiting apical dominance, as BA primarily induces the formation of new shoots. While BA primarily induces axillary shoot proliferation and may inhibit apical dominance, 2-iP exerts a more marked effect on shoot elongation (Al-Sulaiman and Barakat 2010). Lemongrass plants cultured on a medium containing 10 µM BAP exhibited a typical cytokinin response, producing an average of 23.3 shoots per explant after two months. In contrast, when 2-iP was applied at the same concentration under identical conditions, a significantly lower number of shoots (8.8 shoots per explant) was observed, indicating an inverse relationship between the effects of BAP and 2-iP on shoot proliferation in this species (Cárdenas-Aquino et al. 2023). The superiority of BA over 2-iP in enhancing shoot multiplication has also been reported in previous studies, including Ahmed and Anis (2014) in *Vitex trifolia*, and Ahmad et al. (2022) in *Casuarina equisetifolia*.

Among various cytokinin treatments (2-iP, BA, and KIN), 2-iP was the most effective in promoting shoot elongation, yielding the highest mean shoot length per explant in *Dianthus* species (Farkas et al. 2025). These findings agree with previous studies that reported enhanced shoot elongation in response to 2-iP treatment. Guang-jie et al. (2008) demonstrated that 2-iP influences shoot height, with the increase in shoot height proportional to the increasing concentration of 2-iP in the culture medium. The results of this study are consistent with previous research on *Hymenocallis littoralis*, confirming that 2-iP is a more effective cytokinin than BA in promoting shoot elongation (Chai et al. 2010). Jana et al. (2013) also reported that among four cytokinins studied on *Sophora tonkinensis*, shoot height was highest in the medium supplemented with 2-iP.

#### Effect of NAA and IBA on the in vitro *E. asperula* root induction

The formation of adventitious roots is regulated by both endogenous and exogenous factors. Among the internal factors, phytohormones, particularly auxins, are considered to play a central role. Auxins are widely recognized for initiating root formation (Štefančič et al. 2005). Auxins regulate various plant growth and development aspects, including lateral root initiation and root gravitropic responses. Numerous studies have shown that the application of exogenous auxins significantly enhances lateral root formation, and both root initiation and development are strongly dependent on auxin concentration and its polar transport (Chhun et al. 2003). Previous studies have demonstrated that auxins such as IAA, IBA, and NAA facilitate the induction and development of adventitious roots in *Chrysanthemum morifolium* and *Mentha piperita* L. (Imtiaz et al. 2019; Islam et al. 2017). However, the specific effects of these auxins differ among plant species, likely due to variations in auxin receptor affinity and downstream signalling pathways involved in root organogenesis (Huang et al. 2022).

The role of auxins, particularly NAA, has been extensively investigated in several plant species, including *Malus species* (De Klerk et al. 1997), *Centaurea tchihatcheffii* (Ozel et al. 2006), and *Oryza sativa* (Chhun et al. 2003). In particular plant species, high auxin concentrations have been shown to inhibit root formation, often leading instead to callus development at wounded sites. Previous studies have reported that NAA is effective for root induction only at low concentrations. For instance, Yan et al. (2014) reported that lower concentrations of NAA significantly promoted adventitious root formation in *Hemarthria compressa*, and similar results were observed by Kaur et al. (1999) in *Valeriana jatamansi*. In the case of *E. asperula*, callus formation at the shoot base was observed after 4 weeks of culture on NAA-supplemented medium. The callus formation frequency reached 100%, suppressing adventitious root induction in the in vitro shoots (Figure 10I). Histological examination of the callus tissue at the shoot base revealed no evidence of root primordia formation from the stem (Figure 10J). These findings indicated that NAA is unsuitable for inducing root formation in this species, even when applied at low concentrations (the lowest concentration used in this study was 0.5 mg l^−1^ NAA).

**Figure 8:**
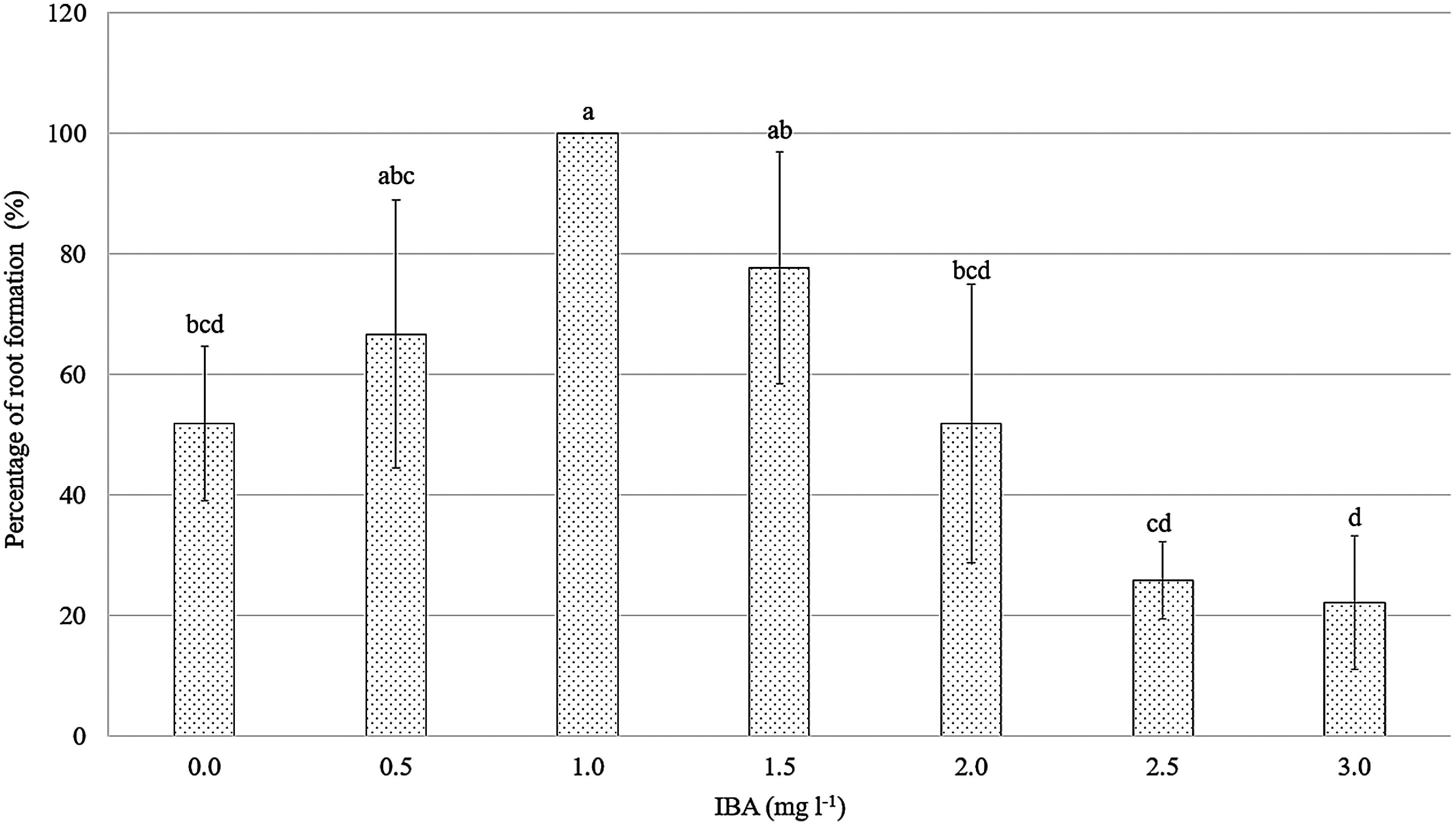
Figure 8. Effect of the IBA concentration on the percentage of root formation. Means followed by different letters indicate significant differences according to Tukey’s HSD test (*p*<0.05).

**Figure 9:**
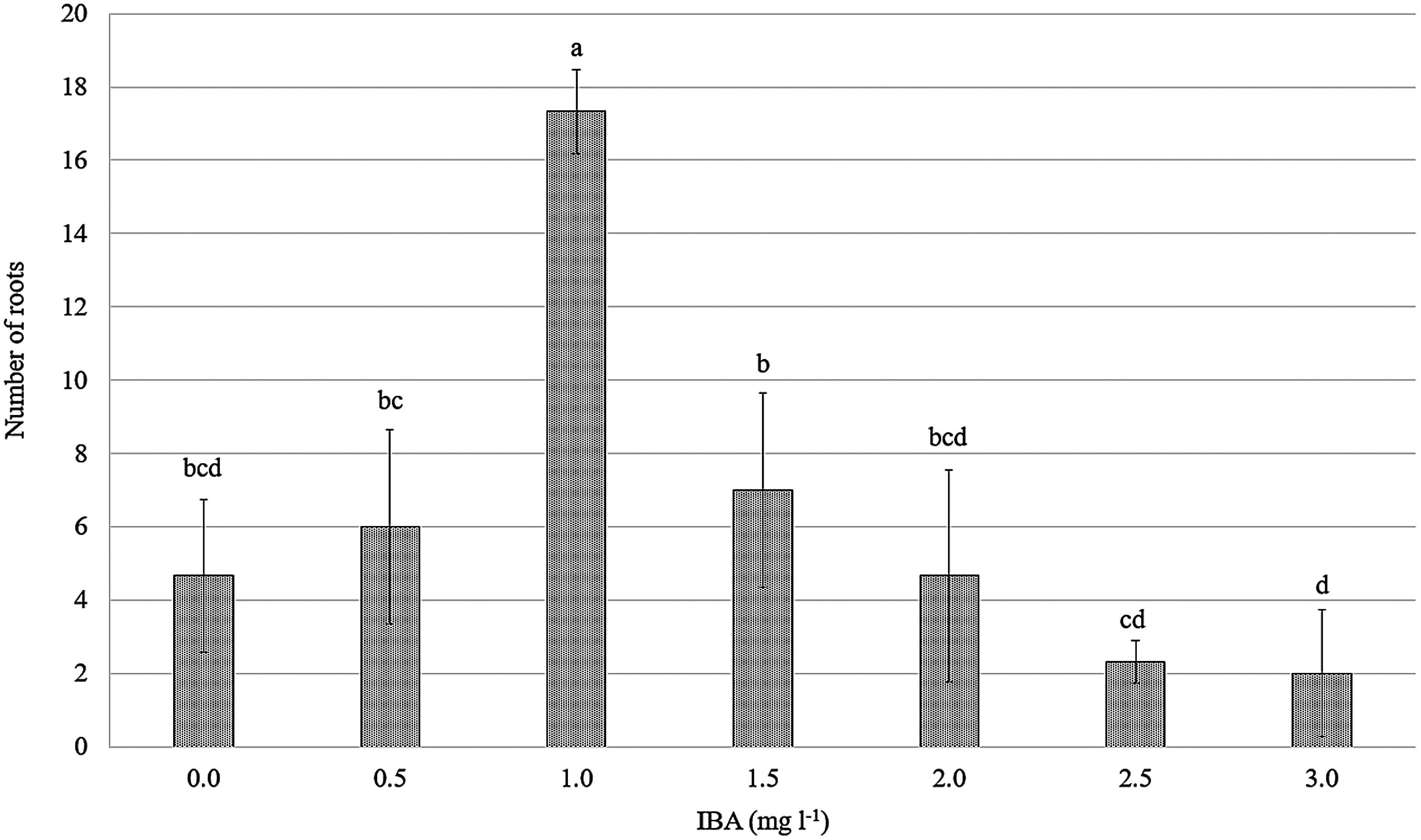
Figure 9. Effect of the IBA concentration on the number of roots. Means followed by different letters indicate significant differences according to Tukey’s HSD test (*p*<0.05).

**Figure 10:**
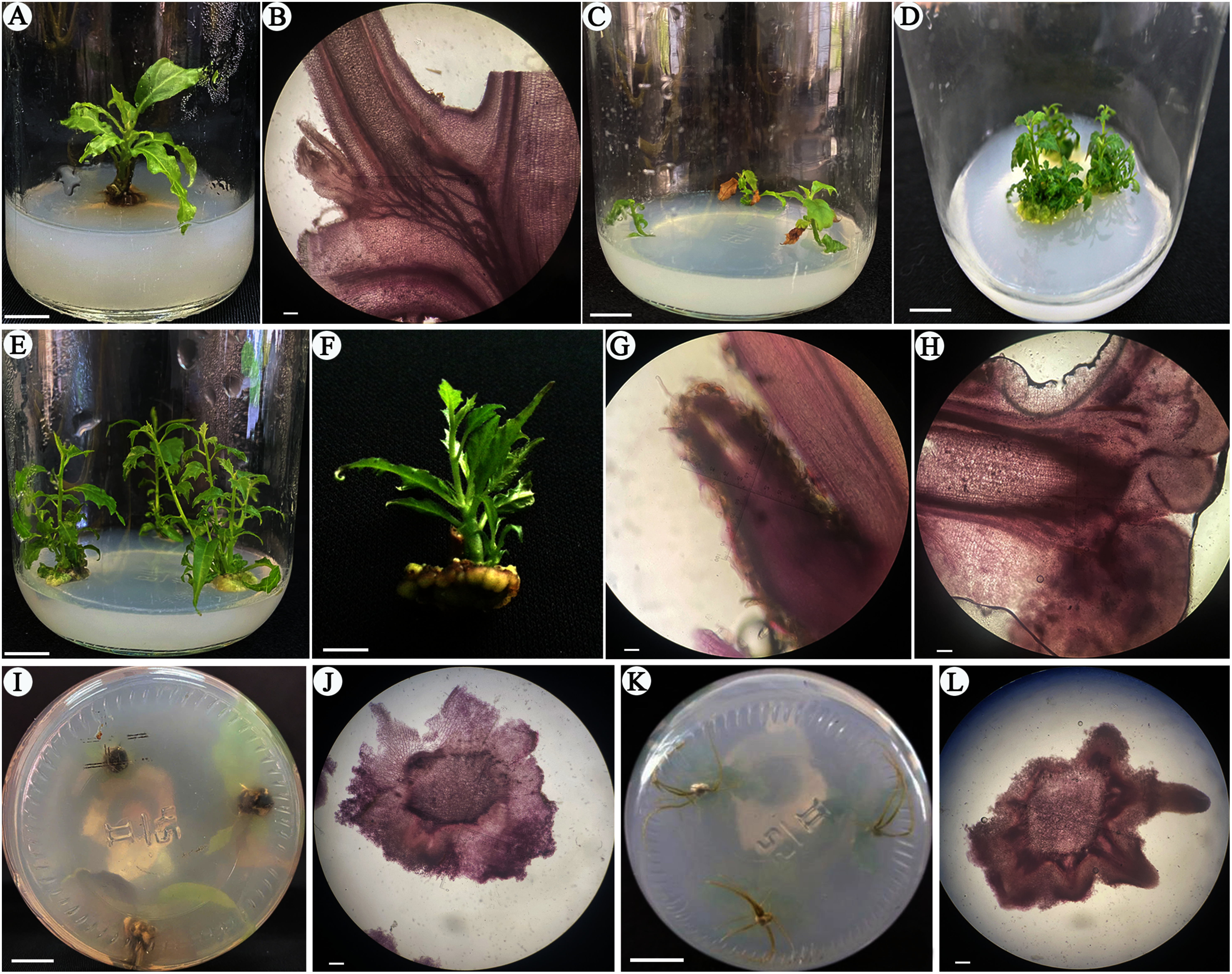
Figure 10. Effect of plant growth regulators on the shoot and root development in *E. asperula* after 4 weeks of culture; Scale bars: A, C–F, I, K=1.0 cm; B, G, H, J, L=10 µm; A, The shoot regeneration from the node explants on the MS with 3.0 mg l^−1^ KIN; B, The anatomical morphology of axillary shoot meristem; C, D, E, Shoot proliferation on the MS medium without and with 3.0 mg l^−1^ BA or 1.5 mg l^−1^ 2-iP; F, In vitro shoot regenerated from callus; G, The anatomical morphology of shoot apical meristem; H, The anatomical morphology of shoot regeneration from callus; I, Callus formation on media supplemented with NAA; J, Anatomical morphology of the callus; K, Root formation on medium supplemented with 1.0 mg l^−1^ IBA; L, Anatomical morphology of in vitro root development.

In contrast to NAA, all *E. asperula* shoots cultured on media supplemented with IBA exhibited root formation after four weeks of in vitro culture (Figure 10K). The root growth parameters varied depending on the IBA concentration (*p*<0.05, Figure 8, 9). The highest rooting response was observed in explants cultured on MS medium supplemented with 1.0 mg l^−1^ IBA, with a rooting rate of 100%, an average of 17.33 roots per explant. These values were higher than those recorded in other IBA treatments and the control. However, increasing the IBA concentration from 2.0 to 3.0 mg l^−1^ inhibited root formation, with the rooting percentage decreasing from 51.85% to 22.22% and the number of roots per explant dropping to 4.67–2.00. Alsemaan (2013) reported that the rooting percentage reached 83% when shoots were cultured on medium supplemented with 2.0 mg l^−1^ IBA; however, root quality decreased at higher concentrations of IBA, and fewer roots were formed. The application of exogenous auxins in plant tissue culture alters the levels of endogenous auxins within the plant, thereby affecting the induction and development of roots (Ribnicky et al. 1996). According to Justamante et al. (2019), auxin is one of the key endogenous hormones involved in adventitious root formation, and the physiological stages of rooting are correlated with changes in endogenous auxin levels. Several previous studies have demonstrated the effectiveness of IBA in inducing root regeneration in various plant species, such as *Prunus dulcis* Mill, and *Bougainvillea glabra* ‘New River’ (Kodad et al. 2021; Lin et al. 2024).

When comparing the root induction efficiency of two auxins, NAA and IBA, in in vitro cultured *E. asperula*, the results showed that IBA was significantly more effective in promoting root formation. This finding is consistent with the study by Deng et al. (2015) in *M. officinalis*, in which among the three auxins tested, IBA more effectively promoted root formation of in vitro shoots compared to NAA. Root formation from in vitro shoots plays a crucial role in the micropropagation process, as this stage determines the survival rate of seedlings after transfer to the nursery. Therefore, identifying the appropriate type and concentration of auxin is essential in the micropropagation of *E. asperula*.

When observing the anatomical morphology (Figure 10L), the shoots cultured on medium supplemented with IBA showed callus formation at the base of the shoots. However, this callus was porous and friable, developing from the stem, and thus did not impede the rooting process. Anatomical morphology revealed that root primordia were initially formed from the stem, followed by elongation and development into a complete root system.

## Conclusion

*E. asperula* from Hoa Binh exhibited the highest TPC and RosA content, along with the most potent antioxidant activity, making it the most promising source of bioactive compounds. An optimized 30% ethanol and 70% water extraction solvent and a successful micropropagation protocol were established, enabling large-scale production of genetically uniform plantlets for sustainable cultivation and standardized sources of medicinal and cosmetic raw materials.

## Data Availability

All data generated or analyzed during this study are included in this published article.

## Abbreviations

2-iP: 6-(γ,γ-dimethylallylamino)purine
BA: 6-benzylaminopurine
IBA: Indole-3-butyric acid
IC_50_: 50% inhibitory concentration
KIN: kinetin
MS: Murashige and Skoog medium
NAA: α-naphtalene acetic acid
RosA: rosmarinic acid
TFC: total flavonoid content
TPC: total phenolic content

## Accession numbers

PQ237099 (Hoa Binh), PV973038 (Dong Nai); PV973039 (Vinh Phuc).
